# Cockroach a Possible Conveyor of Cancer

**Published:** 1913-11

**Authors:** 


Cockroach a Possible Conveyor of Cancer. The *Lancet,* February 1, 1913, refers to a previous communication by W. Melville-Davison claiming that cancer is due to an alga which is found in the intestine of the cockroach and elsewhere. Febiger of Copenhagen claims that an intestinal worm of the rat passes its larval stage in the intestine of the cockroach and that cancer in the rat is due to his eating the cockroach. We are inclined to doubt any specific convection of cancer by the cockroach, but there is no question but that various infections may be spread adventitiously by this insect which swarms from plumbing, floors and garbage over tooth brushes, towels and food.

